# The first high-quality genome assembly and annotation of *Patiria pectinifera*

**DOI:** 10.1038/s41597-023-02508-1

**Published:** 2023-09-20

**Authors:** Jaehoon Jung, So Yun Jhang, Bongsang Kim, Bomin Koh, Chaeyoung Ban, Hyojung Seo, Taeseo Park, Won-Jae Chi, Soonok Kim, Heebal Kim, Jaewoong Yu

**Affiliations:** 1 eGnome, Inc., 26 Beobwon-ro 9-gil, Songpa-gu, Seoul, 05836 Republic of Korea; 2 Department of Agricultural and Life Sciences and Research Institute of Population Genomics, Seoul National University, Seoul, Republic of Korea; 3 Interdisciplinary Program in Bioinformatics, Seoul National University, Seoul, 151-742 Republic of Korea; 4 Animal Resources Division, National Institute of Biological Resources, Incheon, 22689 Republic of Korea; 5 Microorganism Resources Division, National Institute of Biological Resources, Incheon, 22689 Republic of Korea

**Keywords:** Genome informatics, Comparative genomics

## Abstract

The blue bat star, a highly adaptive species in the East Sea of Korea, has displayed remarkable success in adapting to recent climate change. The genetic mechanisms behind this success were not well-understood, prompting our report on the first chromosome-level assembly of the *Patiria* genus. We assembled the genome using Nanopore and Illumina sequences, yielding a total length of 615 Mb and a scaffold N50 of 24,204,423 bp. Hi-C analysis allowed us to anchor the scaffold sequences onto 22 pseudochromosomes. K-mer based analysis revealed 5.16% heterozygosity rate of the genome, higher than any previously reported echinoderm species. Our transposable element analysis exposed a substantial number of genome-wide retrotransposons and DNA transposons. These results offer valuable resources for understanding the evolutionary mechanisms behind *P. pectinifera*’s successful adaptation in fluctuating environments.

## Background & Summary

**Fig. 1:**
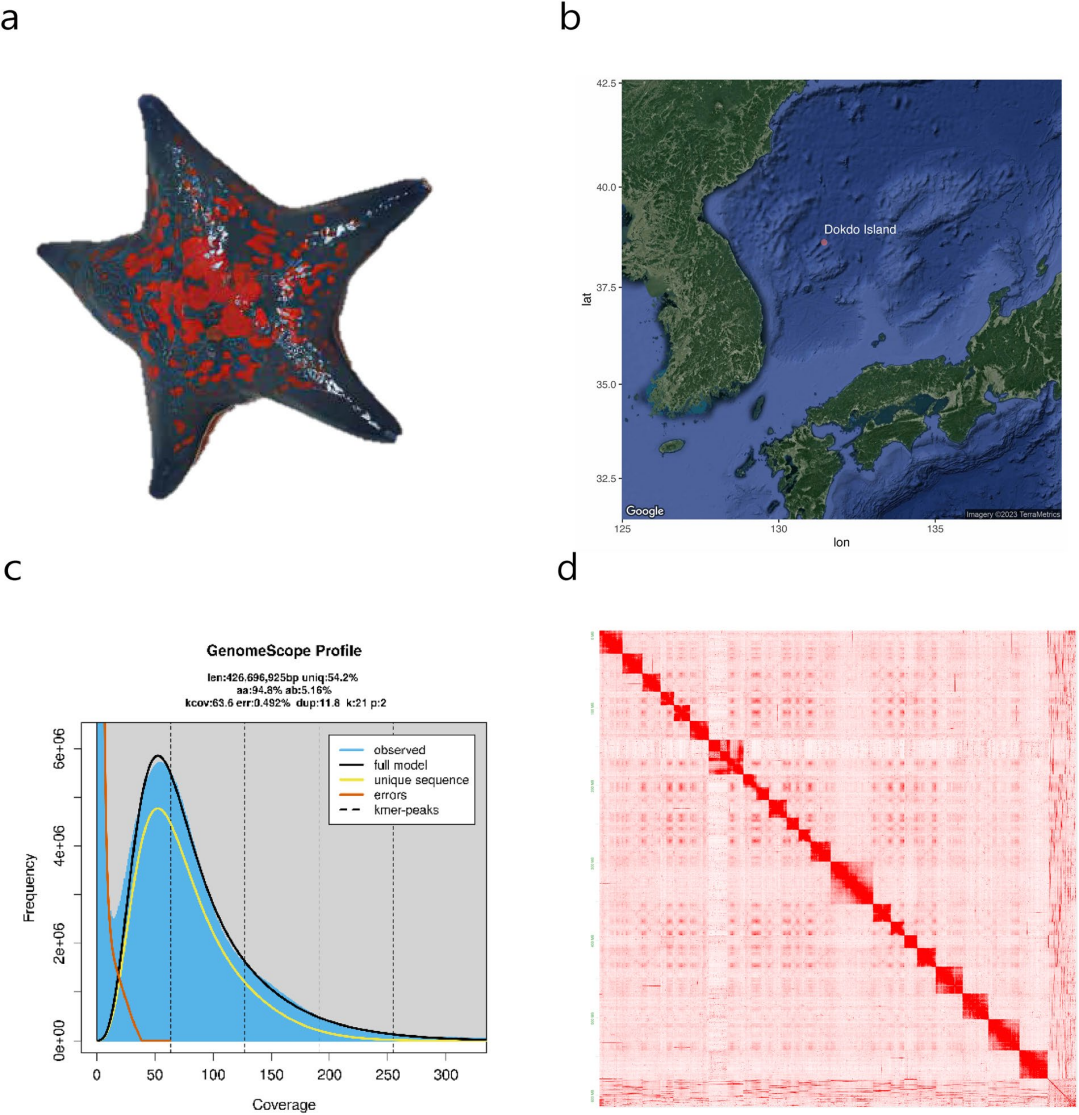
Chromosome-level assembly of *P. pectinifera* genome. (**a**) Image presentation of *P. pectinifera*. (**b**) Geographical origin of the collected sample. The map has been generated by R package “ggmap”. (**c**) K-mer (21-mer) plot of the species for genome size and heterozygosity estimation. (**d**) Hi-C contact map of representing 22 pseudochromosomes.

Adapting to rapidly changing environments is a critical issue in evolutionary biology amidst global warming. The East Sea, known for its extreme seasonal fluctuations in temperature (5–15 °C for the coasts of the Korean Peninsula) and salinity (30~34 permil)^1,2^, is one of the world’s ecoregions most heavily impacted by climate change^3,4^. Among the species inhabiting this region is the blue bat star (*Patiria pectinifera*, Fig. 1a), which thrives in a wide range of environments due to its adaptability and resilience^5^. While recent studies have shown that transposable elements (TEs) can accelerate genetic responses to stress, the underlying mechanisms remain unclear^6,7^.

Besides its adaptation ability, *P. pectinifera* is commonly used as a model organism in developmental biology due to its prevalence, echinoderm’s intermediate phylogenetic position with respect to chordates and protostomes, and ease of maintenance in the laboratory^8–10^. Furthermore, large and transparent oocytes, which remain viable after removal from the gonads, were used for studies of oocyte maturation, fertilization, and larval development^11^. A peptide known as “Gonad Stimulating Substance (GSS)”, which exhibits properties similar to relaxin, has been identified in *P. pectinifera*^12^. This peptide serves as potential evidence of an invertebrate gonadotropin responsible for gamete maturation, exerting its reproductive functions via a G protein-coupled receptor. To date, despite the researchers’ attention and biological importance, the genetic background and evolutionary history of *P. pectinifera* have not been fully understood. There have been studies about tissue-specific antimicrobial peptides (AMPs) gene expression in the species using RT-qPCR and RACE PCR^13^, and researchers have found novel genes and peptides from the species^10,13^. However, its genome has not been constructed thoroughly, needless to say that there was just one whole-genome sequencing dataset for the species in sequence read archive (based on 2023-04-27 release data in SRA, NCBI^14^).

This study strives to advance our understanding of the genetic mechanisms facilitating the successful adaptation of *P. pectinifera* in variable environments. We utilized long-read sequencing data and Hi-C techniques to construct a chromosome-level genome assembly for the species, enabling the analysis of its distinctive genomic feature – high heterozygosity level. We acknowledge that our comparative genome analyses are preliminary and serve as a steppingstone for further research.

Our insights into the genomic features of *P. pectinifera* augment our understanding of the species’ adaptive capabilities and furnish valuable resources for ongoing studies in evolutionary biology. The chromosome-level genome assembly of *P. pectinifera* can act as a baseline for subsequent comparative genomics studies, thereby enhancing our knowledge of the evolution and adaptation of other echinoderm species, even though we acknowledge that in-depth comparative genomic analysis among diverse echinoderm taxa is required for more definitive conclusions.

## Methods

### Sample information and collection

*P. pectinifera* sample was collected from Dokdo Island (38°14′25.1″N 131°52′13.63″E) in June 2019 for genome assembly analysis (Fig. 1a,b).

### DNA library construction and sequencing

The high molecular weight (HMW) genomic DNA was extracted from gonads utilizing PrepGEM universal DNA extraction kit (microGEM, #PUN0100) for the purpose of sequencing with the Oxford Nanopore Technology (ONT) platform. This extraction process, which was adapted from the CTAB method^15^ with a 2% polyvinylpyrrolidone (PVP) (comprising 1% of molecular weight [MW] 10,000 and 1% of MW 40,000) (Sigma-Aldrich, Burlington, MA, USA). Subsequently, DNA concentration was ascertained via the Quant-iT PicoGreen® assay (Invitrogen, Waltham, MA, USA) while the Synergy HTX Multi-Mode microplate reader (Biotek, Rochester, VT, USA) was used to measure the absorbance at 260 nm and 230 nm (A260/A230). The DNA quality was then verified using gel electrophoresis. Short Read Eliminator Kit (Circulomics, Baltimore, MD, USA) was used to eliminate short genomic fragments under 10 kb. The library was prepared using the ONT 1D ligation Sequencing kit (SQK-LSK109, Oxford Nanopore Technologies, Oxford, UK) with the native barcoding expansion kit (EXP-NBD104) following the manufacturer’s protocol. The constructed library was loaded onto a MinION flow cell (FLO-MIN106 R9.4 Flow Cell) (Oxford Nanopore Technologies) and PromethION Flow Cell (FLO-PRO002) (Oxford Nanopore Technologies). Sequencing was performed on a MinION MK1b and PromethION sequencer with MinKNOW software v19.10.1.

As part of the Hi-C library construction procedure, gonad tissue was introduced to 1% formaldehyde to facilitate chromatin fixation. After this, nuclei isolation was conducted following a method^16^. The fixed chromatin was then subjected to treatment with HindII-HF (New England BioLabs, Ipswich, MA, USA), following which the 5′ overhangs were populated with nucleotides and biotin-14-dCTP (Invitrogen). This allowed for ligation of free blunt ends. Subsequent to ligation, the DNA was purified with unligated DNA ends having biotin effectively removed. Fragmentation and size-based selection were performed on the Hi-C DNA. Finally, the ThruPLEX® DNA-seq Kit (Takara Bio USA, Inc, Mountain View, CA, USA) was employed to complete the preparation of the Hi-C library. Fragmentation and size selection were performed to shear the Hi-C DNA. The Hi-C library preparation was performed using the ThruPLEX® DNA-seq Kit (Takara Bio USA, Inc, Mountain View, CA, USA).

To sequence with the Illumina platform, genomic DNA was randomly sheared using the Covaris S220 system. The fragmented DNA was utilized for library preparation using the TruSeq DNA PCR-Free kit (Illumina, San Diego, CA, USA), adhering to the manufacturer’s instructions. Whole-genome shotgun sequencing was performed using Illumina NovaSeq. 6000 with a 150 bp paired-end. Also, the prepared Hi-C library was sequenced with the same platform. All the obtained reads were quality controlled by trimming adaptor sequences and low-quality reads using Trimmomatic v0.39^17^ for Illumina reads and Porechop v0.2.4 (-q 7 option, https://github.com/rrwick/Porechop) and NanoFilt^18^ (-k 5000 option) for Nanopore reads.

### RNA library construction, sequencing and transcriptome construction

Total RNA was extracted from three distinct tissues - the digestive gland, gonad, and stomach of a *P. pectinifera* individual. This extraction was done using TRIzol Reagent (Invitrogen, Waltham, MA, USA) as per the guidelines provided by the manufacturer. The total RNA concentration was then gauged using the Quant-iT™ RNA Assay Kits (Invitrogen), and the absorbance at 260 nm and 280 nm (A260/A280) was quantified using the Synergy HTX Multi-Mode microplate reader (Biotek, Rochester, VT, USA). The complementary DNA (cDNA) library was then constructed using the cDNA-PCR Sequencing Kit (SQK-PCS109, Oxford Nanopore Technologies), alongside the PCR Barcoding Kit (SQK-PBK004, Oxford Nanopore Technologies), again adhering to the manufacturer’s protocol. All three tissue samples underwent sequencing in the same flow cell using the same procedure as the one employed for the previously mentioned DNA sequencing. We also used the Illumina platform to generate high-quality short reads. Using the Truseq Stranded mRNA Prep kit, we constructed the cDNA library and sequenced it in the Illumina NovaSeq. 6000 with 100-bp paired-end reads.

For transcriptome assembly, filtered long-read RNA sequencing data from 3 tissues were error-corrected with short reads using TALC software^19^. The TALC-corrected reads were aligned to the assembled genome using minimap2 splice-aware option^20^, alignment data were sorted with Samtools v1.9^21^, and reference-based transcriptome assembly was performed using StringTie2^22^.

### Genome Size Estimation and heterozygosity estimation using short read data

The filtered high-quality short-read sequencing data were used to calculate 21-mer distribution through Jellyfish v2.3.0 program^23^. From the jellyfish output, genome size, repeat content, and heterozygosity were estimated using GenomeScope2 Program^24^ at 426 Mb, 45.8%, and 5.16%, respectively (Fig. 1c).

### High-quality genome construction

We utilized NextDenovo v2.4.0 (https://github.com/Nextomics/NextDenovo) to assemble the *P. pectinifera* genome, using a total of 25.95 Gb data in Nanopore sequences with N50 21 kb. After the assembly, assembled contigs were polished with Illumina short reads using four times of NextPolish v1.1.0^25^. Then we employed the Hi-C technology to obtain chromosome-level genome assembly. Firstly, the paired-end Illumina reads were mapped onto the polished assembly using HiC-Pro v3.0.0^26^ with default parameters to check the quality of the raw Hi-C reads. Then Juicer v1.6^27^ and 3D-DNA v180419^28^ were applied to cluster the genomic contig sequences into potential chromosomal groups. Afterward, contig orientations were validated and ambiguous fragments were removed with manual curation using Juicebox v1.13^29^, whereby consecutive contigs were linked to generate a high-quality genome assembly (Fig. 1d). the final chromosome-level genome assembly was created with the largest 22 pseudo-chromosomes, which correspond to the known karyotype of the species^30^ (hereafter referred to as **“Chromosomes”**), and 1,458 small scaffolds with a length of 615 Mb (Table 1).

**Table 1:** Genome Assembly statistics of *P. pectinifera*.

Statistics	Value
Number of Scaffolds	22 (+1458)
Genome Length	579,098,568 (+36,262,640)
Min bp in pseudochromosomes	15,291,986
Max bp in pseudochromosomes	54,886,374
Average bp of pseudochromosomes	26,322,662.18
N50	24,204,423
QV	31.77 (0.00066 error rate)

### Repeat annotation

The identification of repetitive regions in the genomes was accomplished through a two-step process involving ab-initio prediction and homology-based repeat search. Firstly, *ab-initio* prediction approach was accomplished by using RepeatModeler v2.0.1^31^ with RECON v1.08^32^, RepeatScout v1.0.6^33^, rmblast v2.10.0 (http://www.repeatmasker.org/RMBlast.html) and TRF v409^34^ to predict interspersed repeats. With ‘–LTRStruct’ option in Repeatmodeler, LTRs were also identified using LtrHarvest^35^ (on genometools-1.5.9^36^), Ltr_retriever v2.9.0^37^, MAFFT v7.407^38^, CD-HIT v4.8.1^39^, and Ninja v1.2.2^40^. This library, in addition to RepBase^41^, was then utilized to perform a homology-based repeat search throughout the genome using RepeatMasker^42^.

Given the large amounts of unclassified elements observed in our data (89.36% of all repeats), we conducted an additional transposon classification analysis using RFSB tool^43^. This was done to further elucidate and identify the unidentified repeat sequences (Table 2).

**Table 2:** Repeat annotation results comparison.

	Detection Tool		Repeatmasker	Repeatmasker +RFSB
	Total		281,711,337	
Class I	LTR	Gypsy	6,061,638	36,080,823
		Copia	169,828	6,893,215
		others	3,383,226	3,680,142
	Non-LTR	LINE	10,121,821	10,532,230
		SINE	147,399	419,396
Class II		Tc1-Mariner	420,985	7,654,995
		Zator	29,369	57,071,440
		hAT	437,019	126,194,425
		CMC	347,430	17,325,589
		others	2,767,384	9,242,887
Helitron			975,329	1,513,271
Simple repeat			4,188,228	4,188,228
Low complexity			585,869	585,869
Others (Include unknown)			252,075,812	328,827

### Gene prediction and functional annotation

A total of four rounds of genome annotation were performed with MAKER3 pipeline^44^. Initial evidence-based annotation was performed using exonerate v2.4.0^45^, with proteome evidences (*A. rubens*: GCF_902459465.1, *A. planci*: GCF_001949145.1, *P. miniata*: GCF_015706575.1, *S. purpuratus*: GCF_000002235.5, and *P. pectinifera*: 26 proteins from UniProt database^46^) and reference-based transcriptome assembly data. The first round was used to train the gene prediction software SNAP^47^ with the alignment above. In the next round, trained SNAP gene models were used for *ab initio* MAKER3 annotation. Then, the SNAP gene model was trained with second annotation data, and the augustus/genemark gene model constructed from braker2^48^ with transcript alignment data and protein data was used as a third-round annotation. The final round was performed with the EVM module weights of 8, 2, and 1, respectively, to the transcript, protein, and *ab initio* evidence. As a result, 40,468 genes were predicted and the gene structure annotation, predicted CDS and protein sequences and annotation of repeats were uploaded in figshare (See Data Records).

## Data Records

The final genome assembly has been deposited at GenBank with accession number JASAOE000000000^49^. and raw sequencing data from the Nanopore, Illumina, and Hi-C libraries have been deposited at NCBI with accession numbers SRR24423632, SRR24423633, SRR24423634 under BioProject SRP435816^50^.

The transcriptome data of 3 tissues were deposited with accession numbers SRR24423629, SRR24423630, SRR24423631 under the same Bioproject.

All results from genome annotation are available in figshare^51^.

## Technical Validation

### Evaluation of the genome assembly

To evaluate the quality of the genome assembly, we first assessed the completeness of the assembled genome using BUSCO v3.0.2b^52^. The final genome assembly yielded a Complete BUSCO score of 98.5% (Table 3). Second, we measured the Base Accuracy QV value using Merqury^53^, resulting in a QV value of 31.77 (0.00066 error rate) (Table 1). In addition, we computed the N50 and L50 metrics for the genome, which turned out to be 24,204,423 and 11 respectively (Table 1). These quality assessments suggest a high-quality genome assembly.

**Table 3:** Genome assessment of complete genome.

Genome	*P. pectinifera*
Results	C:98.5% [S:96.4%, D:2.1%], F:0.4%, M:1.1%, n:954
Complete BUSCOs (C)	940
Complete and single-copy BUSCOs (S)	920
Complete and duplicated BUSCOs (D)	20
Fragmented BUSCOs (F)	4
Missing BUSCOs (M)	10
Total BUSCO groups searched (n)	954

## Data Availability

All procedures and workflows employed in data processing adhered to the guidelines and protocols outlined in the respective bioinformatics software manuals. This study did not involve the development of any specialized code.
